# Species distribution models of two critically endangered deep-sea octocorals reveal fishing impacts on vulnerable marine ecosystems in central Mediterranean Sea

**DOI:** 10.1038/s41598-017-08386-z

**Published:** 2017-08-14

**Authors:** V. Lauria, G. Garofalo, F. Fiorentino, D. Massi, G. Milisenda, S. Piraino, T. Russo, M. Gristina

**Affiliations:** 1 Institute for Coastal Marine Environment (IAMC), National Research Council (CNR), Via L. Vaccara n 61, Mazara del Vallo (TP), 91026 Italy; 2 Department of Biological and Environmental Sciences and Technologies, University of Salento, 73100 Lecce, Italy; 3 CONISMA - Consorzio Nazionale Interuniversitario per le Scienze del Mare, 00196 Rome, Italy; 4 Department of Economics and Finance, Faculty of Economics, University of Rome Tor Vergata, Rome, Italy

## Abstract

Deep-sea coral assemblages are key components of marine ecosystems that generate habitats for fish and invertebrate communities and act as marine biodiversity hot spots. Because of their life history traits, deep-sea corals are highly vulnerable to human impacts such as fishing. They are an indicator of vulnerable marine ecosystems (VMEs), therefore their conservation is essential to preserve marine biodiversity. In the Mediterranean Sea deep-sea coral habitats are associated with commercially important crustaceans, consequently their abundance has dramatically declined due to the effects of trawling. Marine spatial planning is required to ensure that the conservation of these habitats is achieved. Species distribution models were used to investigate the distribution of two critically endangered octocorals (*Funiculina quadrangularis* and *Isidella elongata*) in the central Mediterranean as a function of environmental and fisheries variables. Results show that both species exhibit species-specific habitat preferences and spatial patterns in response to environmental variables, but the impact of trawling on their distribution differed. In particular *F. quadrangularis* can overlap with fishing activities, whereas *I. elongata* occurs exclusively where fishing is low or absent. This study represents the first attempt to identify key areas for the protection of soft and compact mud VMEs in the central Mediterranean Sea.

## Introduction

Deep-sea coral assemblages play a significant structural role in marine benthic ecosystems by providing essential three-dimensional habitats for fish and invertebrate communities, acting as biodiversity hot spots and contributing to the maintenance of ecosystem functioning^1–3^. They have long lifespans and slow growth rates (few to several mm per year^4, 5^, long reproductive cycles and low recruitment^6^). Their life history traits make deep-sea corals highly vulnerable to human-induced impacts (e.g. fishing, pollution, coastal development, invasive species and climate change). Consequently, a lack of management to preserve deep-sea corals may have permanent or irreversible effects on the entire marine ecosystem^7^.

Deep-sea fisheries, in particular bottom trawling, have dramatic impacts on deep-sea communities as they remove most of the habitat forming organisms from the seafloor^8, 9^ and alter its morphology and physical proprieties (e.g. increased resuspension of bottom sediment, which damages filter feeding organisms by siltation^10, 11^). The impact of fisheries on deep-sea habitats has been increasing over the last decades in response to the decline of many shelf commercial stocks^11, 12^. As a result the abundance of deep-sea corals has been diminishing^13^. The recovery of deep-sea corals from the damage produced by bottom trawling could take decades or centuries, with considerable effects on the associated fish and invertebrate communities and ecosystem biodiversity. International organisations^14–16^ as well as the European Marine Strategy Framework Directive^17^ recognized the fragility of deep-sea habitats, recommending the protection of deep-sea coral species and habitats particularly because they are indicators of vulnerable marine ecosystems (VMEs)^18^. Several deep-sea VMEs have been identified by the General Fisheries Commission for the Mediterranean Sea (GFCM)^19^, however most of these habitats still lack a comprehensive ecological characterisation, including spatial distribution maps.

Important Mediterranean deep-sea coral habitats are represented by relatively dense aggregation of scleractinian colonies of *Lophelia pertusa*, *Madrepora oculata*, *Desmophyllum dianthus* (creating three-dimensional habitats for a number of associated fish and invertebrates taxa), a soft-mud facies characterized by the sea pen *Funiculina quandrangularis* (commonly inhabited by commercially valuable crustaceans such as *Parapenaeus longirostris* and *Nephrops norvegicus*), and a compact-mud facies characterized by the so-called bamboo coral, the gorgonian *Isidella elongata* (associated with high densities of the red shrimps *Aristeus antennatus* and *Aristaeomorpha foliacea* ^20–22^). Due to the high commercial value of the associated crustaceans, the impact of fisheries, and in particular bottom trawling, on Mediterranean deep-sea coral habitats has been severe^22, 23^, and some local key species, such as *I. elongata* and *F. quadrangularis* have been almost entirely eradicated with drastic consequences for the associated invertebrate fauna^10, 22, 24, 25^. Despite the banning of trawling activities below a depth of 1000 m in the Mediterranean by the GFCM for the protection of benthic habitats^26^, the effectiveness of this measure is still unknown. It is certainly inadequate for the protection of several habitat-forming taxa such as *I. elongata* and *F. quadrangularis*, which are partially or exclusively found at shallower depths (see Table 1). Due to such threats adequate marine spatial planning is essential for the conservation of these important habitats in the Mediterranean Sea.

**Table 1:** Information relative to the biology and fisheries of *Funiculina quadrangularis* and *Isidella elongata*.

Species	Description	Anchoring structure	Body flexibility	Habitat type	Fisheries	References
*Funiculina quadrangularis* (Pallas, 1766)	• It is a tall, narrow sea pen, often found in large populations	• A bulb, or peduncle at the bottom of the modified axial polyp that may penetrates into the sediment down to about 50 cm	• It can lie flat under the pressure of wave of approaching gears	• It prefers soft muddy habitat at depths of between 20–2000 m	• This is considered one of the most sensitive sea pen species to fisheries as it is unable to withdraw into the sediment.	22, 75, 83, 84
				• It is often found in moderately high energy environments characterised by a noticeable bottom current, necessary for procuring adequate food		
	• It can reach 2 m in height with the lower quarter embedded in the sediment and usually curved in the upper third					
					• In the northeast Atlantic and Mediterranean Sea is associated with *Nephrops norvegicus* fisheries	
	• It has a calcareous white axis, typically square in section					
	• Information on its longevity does not exist					
*Isidella elongata* (Esper, 1788)	• This species is a near-endemic deep-water gorgonian also known as bamboo coral	• A lobed, root-like holdfast attached to small stones embedded in the surface sediment	• Not flexible due to its fan body shape	• It prefers compact mud	• In the Mediterranean Sea it is usually associated with the high-value, deep-water red shrimps *Aristeus antennatus*, *Aristaeomorpha foliacea* and *Plesionika martia*	72, 85–90
				• This species occurs mainly in intermediate and deep waters (200–1500 m depth) on moderately flat bottoms		
	• It can reach up to 3 m in height forming large single-species stands					
				• This species characterises a facies of bathyal compact mud substrate on moderately flat bottoms (slope <5%)		
	• It is a long-lived species (75–126 years)					

**Figure 1:**
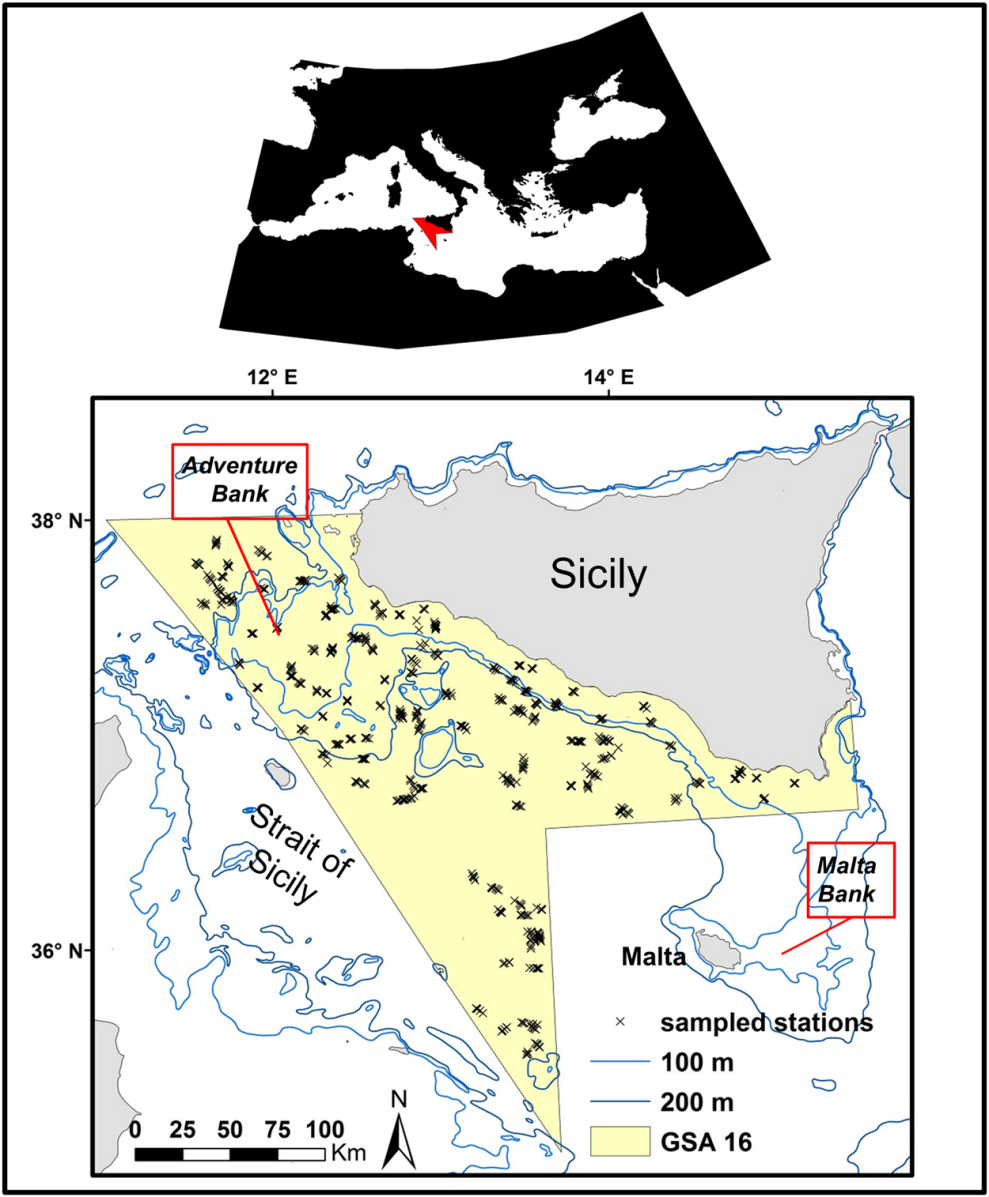
Location of the study region within the Strait of Sicily (Central Mediterranean Sea). This area corresponds to the Geographic Sub Area (GSA) 16. Trawl stations sampled during the MEDITS Survey (2008–2013) are indicated with an x. This map was created with ArcGIS version 10.3 http://www.esriitalia.it by Valentina Lauria.

The central Mediterranean Sea (Figure 1) is a very important area for deep-sea corals, such as white corals (*L. pertusa* and *M. oculata* ^27, 28^). However, little information is available about the large-scale spatial distribution and habitat preferences of other important species such as *F. quadrangularis* and *I. elongata* ^28^ now recognized as critically endangered by IUCN^29^. This area is one of the most important fishing grounds in the Mediterranean Sea, in particular for trawlers targeting deep-water rose shrimp (*P. longirostris*) and other decapods of commercial interest (*A. foliacea*, *N. norvegicus* and *A. antennatus* ^30, 31^). Investigations into the habitat requirements and spatial patterns of these exploited crustacean species are mandatory to ensure their stock management and conservation, as well as the protection of their associated VMEs.

Species distribution models (SDMs) have been increasingly used amongst conservation biologists, ecologists and government bodies to identify where VMEs could occur at regional and global scales and to provide insight into the environmental drivers that control their distribution^32, 33^. The main advantages of using such models is the ability to predict the species presence or abundance at unsurveyed locations, to understand species-environment relationships and to provide distribution maps that can be used to inform policy makers (e.g. planning of marine protected areas^34–36^). In this study SDMs were applied to investigate the preferential habitat (portion of potential habitat used on average over time) and fisheries impacts on *F. quadrangularis* and *I. elongata*, two critically endangered deep-sea octocorals in the central Mediterranean Sea. Species densities were extracted from a long-term dataset (covering the period 2008–2013) and modelled as a function of physical (i.e. depth, slope, rugosity, aspect), oceanographic (i.e. sea bottom temperature, sea bottom salinity and currents), and fisheries variables (i.e. average distribution of the fishing effort over time). Predictive distribution maps were produced in order to identify species-specific spatial patterns at a regional scale. This study will provide new knowledge about the habitat preferences and fishing impact on two critically endangered deep-sea octocoral species and will support future spatial conservation plans for VMEs in the Mediterranean Sea.

## Materials and Methods

### Study area

Our study area is situated in the central Mediterranean Sea and includes the northern side of the Strait of Sicily between 34°59′–38°00′N and 10°59′–15°18′W (Fig. 1). This area corresponds to the Geographic Sub Area (GSA) 16 of the GFCM^37^ and extends for about 34 000 km^2^. It presents a varied seafloor morphology, including a shallow bank (Adventure Bank) in the western part (about 100 m depth) and deeper areas in the southeast (about 1800m depth; Fig. 1). It shows a complex circulation pattern that makes the Strait of Sicily a highly productive area^38^ and a biodiversity hotspot for bony fish, elasmobranchs and invertebrates^39–41^. A number of significant deep-sea coral communities are found in this region, mainly dominated by the octocorals such as bamboo coral *I. elongata*, tall sea pen *F. quadrangularis* and red coral *Corallium rubrum* ^28^. Since the early 1980s, this area has been intensively exploited by many demersal fisheries, mainly bottom trawlers, operating along the southern coast of Sicily, including the Mazara del Vallo fleet, one of the largest and most active fleets in the Mediterranean^42^.

### Survey data

Since 1994 the area has been investigated under the Mediterranean International Trawl Survey program -MEDITS^43^. This survey is carried out annually in late spring/early summer, and takes place in several areas of the Mediterranean Sea using a standardised sampling methodology^44^. It provides a spatio-temporal dataset of fishery-independent indices relating to demersal species abundance, demographic structure and spatial distribution. In GSA16, sampling stations are fixed (Fig. 1) and replicated each year according to a stratified random sampling design based on five depth strata: 10–50 m, 51–100 m, 101–200 m, 200–500 m, 500–800 m, where the number of hauls is proportional to the area of each stratum. A total of 120 stations (haul duration = 30–60 min; trawl speed = 3 knots) were sampled for each year of our study period (2008–2013) on board the commercial trawler Sant’Anna. The gear was a bottom trawl net with a high vertical opening and 20 mm side diamond stretched mesh in the cod-end. At each trawl station the collected species were sorted, weighed, counted and measured. Species density was calculated as the number of individuals per km^2^ (Nkm^−2^) for a total of 720 trawl hauls covering the period 2008–2013. The overall percentage of occurrence (described as the number of hauls in which the species was found) for *F. quadrangularis* and *I. elongata* was calculated. Information about deep-sea octocoral biology and fisheries is provided in Table 1.

### Environmental and fishery variables

**Figure 2:**
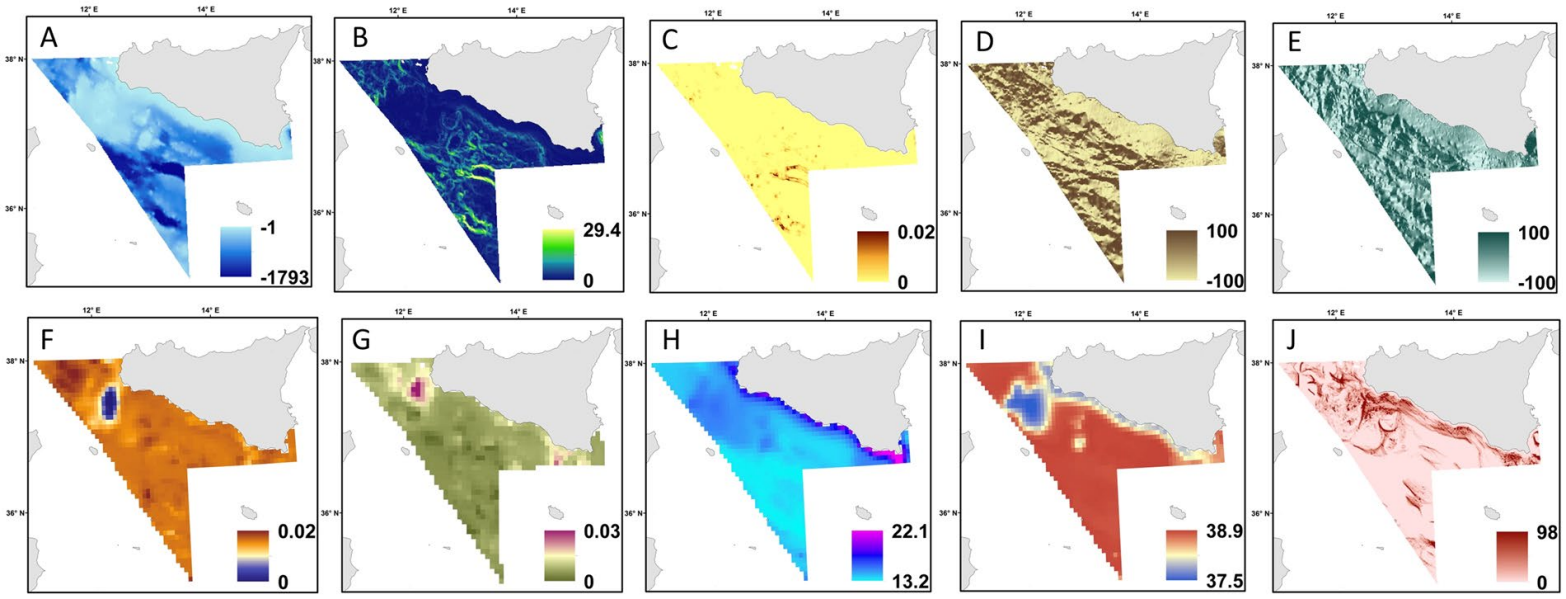
The spatial patterns of the environmental variables used to map the habitat models. These include (**A**) depth (m); **(B**) slope (degrees) values range from to 0° to 90° with low slope values corresponding to flat terrain and higher values to steeper terrain; (**C**) rugosity values range from 0 (no terrain variation) to 1 (complete terrain variation); (**D**) aspect north-south and east-west (**E**) scaled to 100 (radians); (**F**) Current north-south (m/s); (**G**) Current west-east (m/s); (**H**) sea bottom temperature (°C); (**I**) sea bottom salinity (PSU); (**J**) Average distribution of the fishing effort between 2008–2013 in terms of number of fishing hours/year. These maps were created with ArcGIS version 10.3 http://www.esriitalia.it by Valentina Lauria.

For modelling, both environmental and fishery data were used as predictors of octocoral habitat suitability (Table 2; Fig. 2). Environmental variables included physical descriptors (i.e. depth, slope, rugosity and aspect) and oceanographic variables (i.e. sea bottom temperature - SBT, sea bottom salinity - SBS and currents), while for fishery impact predictor the average distribution of the fishing effort over time was used. Digital continuous maps were obtained for all the variables from existing databases, or derivate in ArcGIS (Table 2).

**Table 2:** Predictors used for habitat modelling.

Variable	Unit	Resolution	Descriptor	Type of data	Data source	Reference
Depth	m	0.866 km	Bathymetry	Continuous digital map	http://www.marspec.org/	46
Slope	degrees	0.866 km	Typology of substrata	Derived from bathymetry data	Benthic Terrain Modeller in ArcGIS 10.3	47–49
Rugosity	No unit	0.866 km		Derived from bathymetry data	Benthic Terrain Modeller in ArcGIS 10.3	47–49
Aspect East/West and North/South	Radians	0.866 km	Orientation of the substrata	Derived from bathymetry data	http://www.marspec.org/	46, 51
Currents	m/s	6.5 km	Current	Derived from model	http://marine.copernicus.eu/	91
Sea bottom temperature	°C	6.5 km	Temperature	Derived from model	http://marine.copernicus.eu/	91
Sea bottom salinity	psu	6.5 km	Salinity	Derived from model	http://marine.copernicus.eu/	91
Fishing pressure	Number of fishing hours/year	0.866 km	Fishery effort	VMS data	European Vessel Monitoring System	92

Depth (Fig. 2A) is one of the main environmental gradients that govern species spatial patterns, and in the case of corals has been shown to be a main factor that influences their distribution^20, 32, 45^. Aside of giving information on species bathymetry range this environmental factor can also capture the potential effect of unmeasured variables that might shape species spatial distribution. For this study bathymetry data were extracted from a re-projection of the MARSPEC database, a world ocean dataset developed for marine spatial ecology^46^.

Slope and rugosity provide a measure of the seabed morphology and were derived from the continuous digital map of depth using the Benthic Terrain Modeller tool in ArcGIS 10.1^47^ (Table 2). Slope (Fig. 2B) describes the rate of change in elevation over distance. Low values of slope are associated with flat ocean bottoms or areas of sediment deposition, while higher values indicate potential rocky ledges. Rugosity (Fig. 2C) provides an indicator of the bumpiness and complexity of the seafloor and emphasises small variations in the seabed terrain. Low values of terrain variables indicate soft seabed substrata while high values indicate a rocky seabed. These parameters are largely used as predictors of species distribution when detailed information on sediment type is not available^48, 49^. Studies on the distribution of deep-sea corals have shown different species (i.e. sea pen *Virgularia mirabilis*, *Funiculina quadrangularis* and *Pennatula phosphorea*; gorgonian corals *Paragorgia arborea* and *Primnoa resedaeformis*) preference for a range of sediments and sea bottom types (from soft to hard^45^, some of which might be important for initial coral settlement^50^).

Aspect (Fig. 2D and E; Table 2) identifies the orientation of the seabed at any given location and provides information on the exposure of any given area to local and regional currents^51^. This seabed topographic feature is essential in shaping benthic community structure as it can influence current regimes and the flux of suspended food material^50, 52^. This terrain variable has been shown to be a good indicator of habitat suitability for cold waters corals as it acts as a proxy for other environmental processes^53^. It is measured in radians and divided into two components: North-South and East-West gradient, which vary between −1 and +1 describing the direction that the surface slope faces.

Data on currents, temperature and salinity were obtained from the Ocean General Circulation Model (OGCM) implemented for the Mediterranean Sea. Despite these data have a broader resolution than the bathymetry derived data they represent key environmental drivers of deep-sea corals habitat selection (Table 2). Monthly mean bottom values were extracted from the OGCM model corresponding to the month of the median day of the survey (see online Supplementary Information Table S1). Maps of currents are presented in two directions: eastward and northward (Fig. 2F and G). Bottom currents are an important environmental factor for the habitat selection of deep-sea corals by governing food supply, and the fouling of corals with sediment, as well as influencing larval dispersal and connectivity between suitable habitat patches^54, 55^.

SBT data were also extracted from the OGCM model (Table 2; Fig. 2H). Sea temperature plays a key role in the habitat selection of deep-sea corals, as this environmental factor is thought to influence their calcification rates, physiology and biochemistry^56^. Some studies have suggested that temperature does not limit coral growth and distribution^57^. Data on SBS values were obtained from the OGCM model (Table 2; Fig. 2I). This variable can influence the water column stratification and small oceanographic processes (e.g. internal waves) that may affect food availability^33^.

The fishing pressure exerted by trawlers in the area was used to quantify the potential impact of towed fisheries. The trawling pressure was estimated using the data provided by the vessel monitoring system (VMS), the main geo-positioning device currently used to track and analyse the spatio-temporal behaviour of fishing vessels. The VMS data covering the years 2008–2013 were processed following the methodology described in^58, 59^ and used to assess the spatial distribution of the fishing effort in terms of number of fishing hours per year (Fig. 2J; Table 2).

Values of environmental and fisheries variables were extracted in ArcGIS per station and then used for model construction.

### Model selection

All variables were tested for collinearity using the Variance Inflation Factor^60^ and used for habitat model construction. Histograms of species densities showed a discontinuity between the zero values and positive density data, therefore a two-part modelling approach seemed appropriate^61^. In the case of trawl samples, due to both trawl geometry and species behaviour, zero observations may indicate either low density or true absence, with different processes governing presence probability and density levels^62^. Generalised Additive Models (GAMs) were used to construct a two-part model consisting of a binomial occurrence model developed using presence-absence data (family binomial and logit link function) then modelling presence-only data (positive log-transformed densities) using a Gaussian distribution with a canonical (identity) link function. The final preferential habitat model (also known as delta model) was obtained by the multiplication of the predictions from both models. GAMs are non-parametric regression techniques^63^ that allow for the modelling of relationships between variables without specifying any particular form for the underlying regression function. The use of smooth functions as regressors gives GAMs greater flexibility over linear (or other parametric) types of models.

Starting from the full model that included all explanatory variables, the most parsimonious model was selected on the basis of the lowest Akaike Information Criterion (AIC), corrected for small sample size (AICc). This approach selects the model with the best balance between bias and precision and avoids problems of, for example, multiple testing among explanatory variables^64^. All possible models (set of candidate models that included all combinations of explanatory variables) were ranked using the difference in AICc between the top-ranked and current model (delta AICc), and by calculating the AICc weight (the scaled likelihood that each model is the best description of the data). Competing models of the best supported model were selected when having their AICc within 2 of the minimum^64^. In addition the relative importance of the predictor variables was also calculated by summing the AICc weights from the models that contain that variable. Model performance was measured as the proportion of the null deviance explained (Dev) or the adjusted regression coefficient (R^2^).

Binomial models were tested for sensitivity by using the area under the receiver operating characteristic curve (AUC)^65^. An AUC value of 0.5 indicates that the model performs no better than a random model, whereas a value of 1 indicates that the model is fully capable of distinguishing between occupied and unoccupied sites. AUC values of 0.7–0.9 indicate very good discrimination, while values >0.9 indicate excellent discrimination. Final models were evaluated by comparing predictions in relation to the observations with Spearman’s rank correlation test (r_s_) corrected for spatial autocorrelation and implemented in SAM software^66, 67^. The predictive power of each final model was qualitatively assessed using a range of diagnostic plots^60^. All modeling was carried out using the mgcv library in R v.3.2.0 software^68, 69^.

### Model Mapping

Maps of species predictions were constructed within the raster and rgdal libraries in R^70^ and visualized in ArcGIS software (version 10.3). The model error, defined as the absolute difference between observed and predicted species abundance, was also used to check and illustrate model fit. The spatial distribution of the model error was scaled between 0 and 1 (with a value of 1 corresponding to the maximum possible prediction error) and mapped by interpolation using the Inverse Distance Weighting (IDW) algorithm (in ArcGIS software). This method allows predicting a value for any unmeasured location as it uses the measured values surrounding the prediction location. IDW interpolation assumes that the measured values closest to the prediction location have more influence on the predicted value than those farther away and each measured point has a local influence that diminishes with distance. It gives greater weights to points closest to the prediction location, and the weights diminish as a function of distance.

## Results

**Figure 3:**
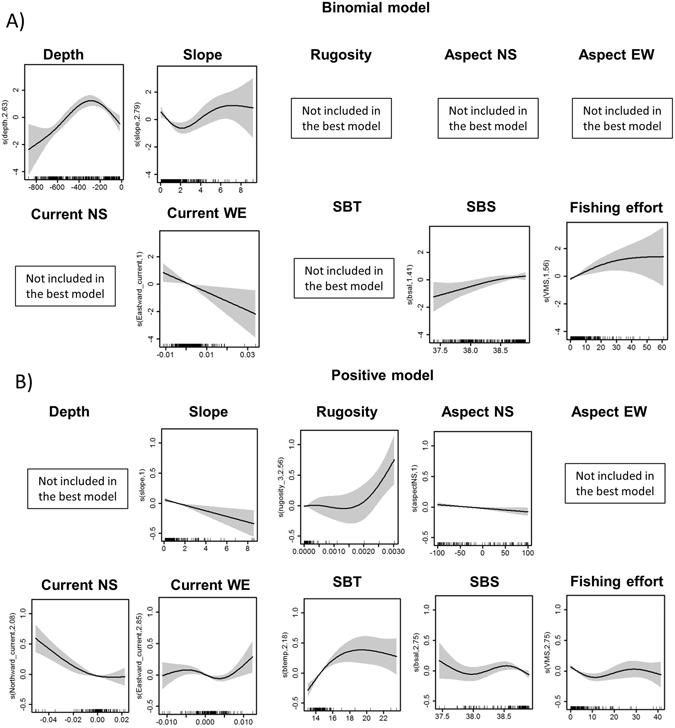
Partial GAM plots for the best binomial and positive models for *Funiculina quadrangularis*. Each plot represents the response variable shape, independent of the other variables, in relation to the probability of the species occurrence (**A**) and abundance (**B**) in the multivariate model. The ranges of environmental variables are represented on the x-axis and the probability of occurrence of the species is represented on the y-axis (logit scale). The zero value indicates mean model estimates, while the y-axis is a relative scale where the effect of different values of the predictors on the response variable is shown. The degree of smoothing is indicated in the y-axis label. Confidence intervals (95%) around the response curve are shaded in grey.

**Figure 4:**
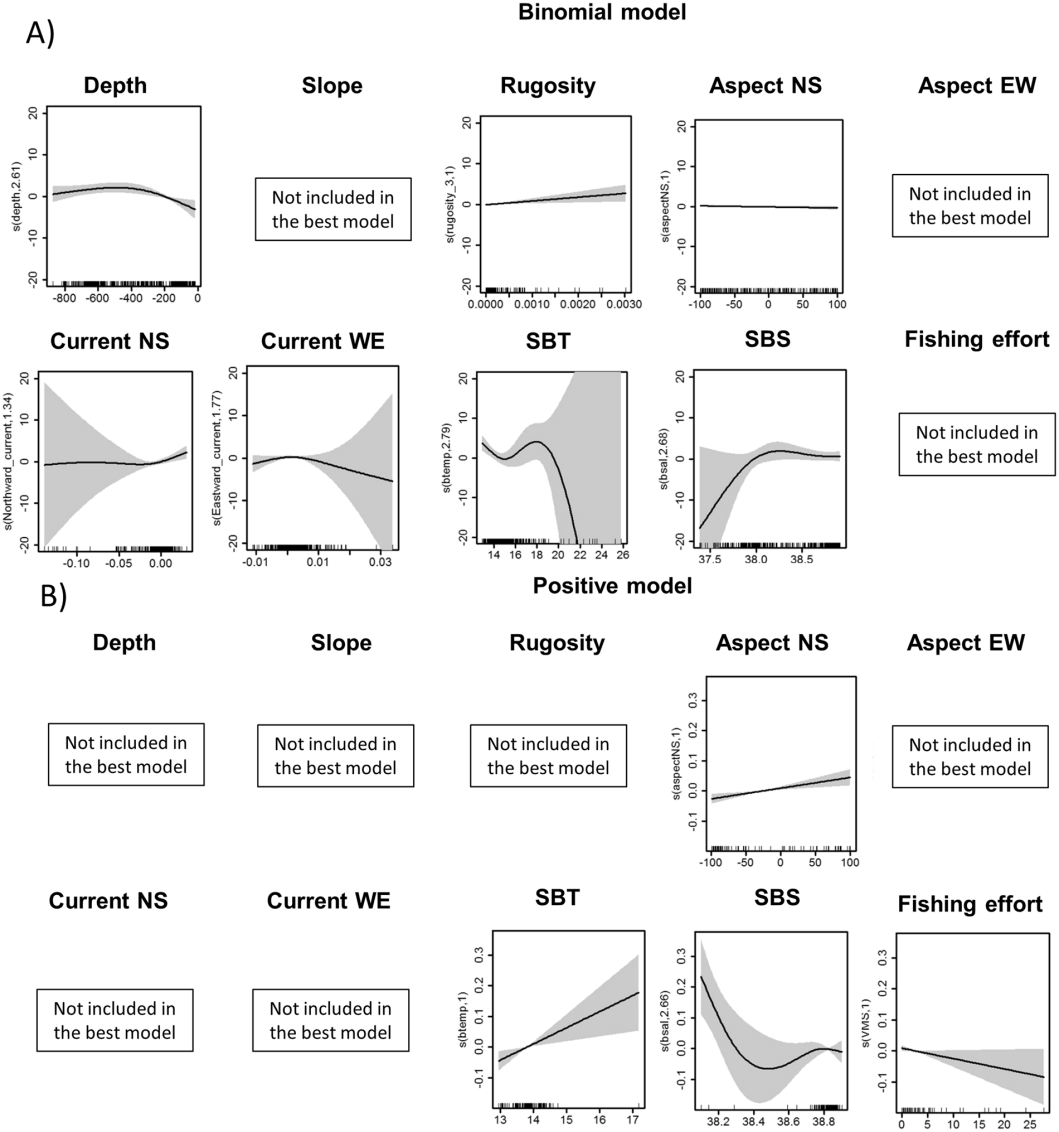
Partial GAM plots for the best binomial and positive models for *Isidella elongata*. Each plot represents the response variable shape, independent of the other variables, in relation to the probability of the species occurrence (**A**) and abundance (**B**) in the multivariate model. The ranges of environmental variables are represented on the x-axis and the probability of occurrence of the species is represented on the y-axis (logit scale). The zero value indicates mean model estimates, while the y-axis is a relative scale where the effect of different values of the predictors on the response variable is shown. The degree of smoothing is indicated in the y-axis label. Confidence intervals (95%) around the response curve are shaded in grey.

The overall percentage of occurrence for *F. quadrangularis* and *I. elongata* was respectively 0.24% and 0.14%. Covariates were not collinear (Variance Inflation Factor <2), models were developed for both species and the results of the best models are summarized in Tables 3 and 4. The relative importance of predictor variables for each species is presented in Table 5. All binomial models passed the sensitivity test suggesting that models had very good discriminating ability (Tables 3 and 4). Depth was found to be an important predictor of presence in both octocoral species habitat models (binomial models only; Tables 3 and 5; Figs 3 and 4). The effect of terrain variables was not particularly pronounced for the habitat suitability of *I. elongata* (rugosity only found in the binomial model, Fig. 4; Tables 4 and 5), while these were important predictors for *F. quadrangularis* (Tables 3 and 5; Fig. 3). Aspect North-South was also an important predictor for the *I. elongata* habitat model, while for *F. quadrangularis* it was found in the positive model only (Table 5; Figs 3 and 4). Conversely, aspect East-West was not significant for any of the coral species habitat models (Tables 3 and 4), still this variable had a modest relative importance for *F. quadrangularis* positive models (Table 5). Our results suggest that the habitat selection of *F. quadrangularis* and *I. elongata* is related to both directions of currents (*I. elongata* binomial model only; Tables 4 and 5). Similarly, SBT and SBS and fishing effort were found to be important environmental factors for both species (binomials and positive models; Tables 3 and 5). Model behaviour, showing relationships between species probability of presence/abundance and environmental and fisheries variables, is presented in Figs 3 and 4. Findings by species are given below.

**Table 3:** Best supported and competing models (using binomial and positive models) for *Funiculina quadrangularis*.

	Model	Depth	Slope	Rugosity	AspectNS	AspectEW	CurrentNS	CurrentWE	SBT	SBS	Fishing effort	ΔAIC	AICw	Adj-R^2^	Dev %	r_s_	ROC AUC
***Best model***	**Binomial**	#####	#####					#####		#####	#####	0	0.08	0.16	15.6	0.29 ***	0.77
	**Positive**		#####	#####	#####		#####	#####	#####	#####	#####	0	0.07	0.61	64.9		
*Competing m1*	*Binomial*	#####	#####			#####		#####		#####	#####	0.5	0.06	0.15	15.6	—	—
*Competing m2*	*Binomial*	#####	#####				#####	#####		#####	#####	1.13	0.04	0.15	15.5	—	—
*Competing m3*	*Binomial*	#####	#####			#####	#####	#####		#####	#####	1.39	0.03	0.15	15.5	—	—
*Competing m4*	*Binomial*	#####	#####					#####	#####	#####	#####	1.78	0.03	0.15	15.5	—	—
*Competing m5*	*Binomial*	#####	#####	#####			#####	#####		#####	#####	1.89	0.03	0.15	16.2	—	—
*Competing m6*	*Binomial*	#####	#####		#####			#####		#####	#####	1.93	0.03	0.15	15.5	—	—
*Competing m1*	*Positive*		#####	#####	#####	#####	#####		#####	#####	#####	0.96	0.04	0.60	63.3	—	—
*Competing m2*	*Positive*		#####	#####	#####	#####	#####		#####		#####	1.25	0.03	0.59	61.9	—	—
*Competing m3*	*Positive*	#####	#####	#####	#####	#####	#####		#####		#####	1.25	0.03	0.59	62.2	—	—
*Competing m4*	*Positive*	#####	#####	#####	#####	#####	#####		#####	#####	#####	1.45	0.02	0.60	63.9	—	—
*Competing m5*	*Positive*	#####		#####		#####	#####		#####		#####	1.7	0.02	0.58	61.4	—	—

Variables included in model are indicated with the symbol #. Predictors include depth, slope, rugosity, aspectNS, aspectEW, CurrentNS, CurrentWE, sea bottom temperature (SBT), sea bottom salinity (SBS) and fishing effort. ΔAIC: delta AIC (difference in AIC between the best model and current model); AICw: Akaike’s Information Criteria (corrected) weights, values range from 0 to 1, and high values indicate strong support for a given predictor. Models were evaluated by R^2^-adjusted coefficient and deviance (Dev): percentage of deviance explained. Only for the binomial model the Receiver Operating Characteristic (ROC) and Area Under the Curve (AUC) were calculated. Significance value of the Spearman’s correlation coefficient (rs) (corrected for spatial autocorrelation) for the delta model is given as ***p value < 0.001, **p value < 0.01, *p value < 0.05.

**Table 4:** Best supported and competing models (using binomial and positive models) for *Isidella elongata*.

	Model	Depth	Slope	Rugosity	AspectNS	AspectEW	CurrentNS	CurrentWE	SBT	SBS	Fishing effort	AICw	ΔAIC	Adj-R^2^	Dev %	r_s_	ROC AUC
***Best model***	**Binomial**	#####		#####	#####		#####	#####	#####	#####		0.13	0	0.29	35.3	0.18	0.89
	**Positive**				#####				#####	#####	#####	0.07	0	0.25	29.9		
*Competing m1*	Binomial	#####		#####	#####		#####		#####	#####		0.12	0.20	0.28	34.7	—	—
*Competing m2*	*Binomial*	#####		#####	#####	#####	#####	#####	#####	#####		0.11	0.30	0.29	35.0	—	—
*Competing m3*	*Binomial*	#####		#####		#####	#####	#####	#####	#####		0.10	0.45	0.28	35.2	—	—
*Competing m4*	*Binomial*	####	#####	#####		#####	#####		#####	#####		0.07	1.21	0.27	34.2	—	—
*Competing m1*	*Positive*				#####		#####		#####	#####	#####	0.04	1.33	0.26	30.6	—	—
*Competing m2*	*Positive*				#####		#####		#####	#####		0.03	1.55	0.23	27.6	—	—

Variables included in model are indicated with the symbol #. Predictors include depth, slope, rugosity, aspectNS, aspectEW, CurrentNS, CurrentWE, sea bottom temperature (SBT), sea bottom salinity (SBS) and fishing effort. ΔAIC: delta AIC (difference in AIC between the best model and current model); AICw: Akaike’s Information Criteria (corrected) weights, values range from 0 to 1, and high values indicate strong support for a given predictor. Models were evaluated by R^2^-adjusted coefficient and deviance (Dev): percentage of deviance explained. Only for the binomial model the Receiver Operating Characteristic (ROC) and Area Under the Curve (AUC) were calculated. Significance value of the Spearman’s correlation coefficient (rs) (corrected for spatial autocorrelation) for the delta model is given as ***p value < 0.001, **p value < 0.01, *p value < 0.05.

**Table 5:** Relative importance of predictor variables (calculated as the sum AIC weights from models that contain that variable within the 95% confidence interval) for *Funiculina quadrangularis* and *Isidella elongata*.

Species	Model		Depth	Slope	Rugosity	AspectNS	AspectEW	CurrentNS	CurrentWE	SBT	SBS	Fishing effort
*Funiculina quadrangularis*	Binomial	Importance	1.00	1.00	0.28	0.29	0.50	0.47	0.83	0.29	0.91	0.90
		N containing models	113	111	43	48	59	64	75	50	81	92
	Positive	Importance	0.59	0.69	0.84	0.66	0.66	1.00	0.48	0.90	0.68	0.94
		N containing models	121	83	117	78	106	156	72	111	89	115
*Isidella elongata*	Binomial	Importance	1.00	0.27	0.83	0.72	0.46	0.99	0.65	1.00	0.91	0.15
		N containing models	61	24	39	34	31	57	39	61	43	23
	Positive	Importance	0.36	0.34	0.35	0.99	0.28	0.37	0.28	0.77	0.99	0.70
		N containing models	109	109	102	229	97	108	96	140	232	124

### Funiculina quadrangularis

This species has a wide bathymetric range in our study area but it is preferentially associated with a depth range of 200–400 m (binomial model only; Tables 3 and 5; Fig. 3). The shape of the smoother of slope shows a linear negative relationship in the positive model, while this negative effect is not marked in the binomial model (non-linear relationship; Tables 3 and 5; Fig. 3). A positive non-linear relationship is found with rugosity (positive model only), however the probability of abundance of *F. quadrangularis* is mainly associated with lower values of this terrain variable (Fig. 3). The habitat preference of this species seems to be negatively correlated with North-South aspect and North-South current direction (linear relationship for aspect and non-linear for current in the positive model), while a negative relationship is shown for a West-East current direction (linear in the binomial model and non-linear in the positive; Fig. 3). Our results suggest that this species prefers bottom temperatures of 14–16 °C (positive model only) and bottom salinity values of around 38.5 PSU (Fig. 3). The effect of fishing effort was not consistent between the two models, in particular a positive non-linear relationship was found in the binomial model, while a negative non-linear relationship with fishing effort was found in the positive model suggesting that higher abundances are associated with low values of fishing activity (between 0 and 10 hours/year; Fig. 3).

### Isidella elongata

This species is found at depths between 200–800 m, with higher presence above 400 m depth (binomial model only; Tables 4 and 5; Fig. 4). The shape of the smoothers for rugosity (binomial model only) and North-South aspect (positive model only) suggests that the probability of presence and abundance of this species increases with higher values of these terrain variables (Table 5; Fig. 4). The bamboo coral seems to prefer areas characterised by greater current (binomial model only, North-South direction), while it reaches its optimum in correspondence of low speed of the West-East current (Fig. 4). Our results suggest that this species is associated with habitats where sea bottom temperature values range is 13–14 °C and bottom salinity values are around 38.7–38.9 PSU (Fig. 4). A negative linear relationship is shown with fishing effort, suggesting a rapid decline of *I. elongata* abundance when fishing activity increases (Table 5; Fig. 4).

### Species distribution maps

**Figure 5:**
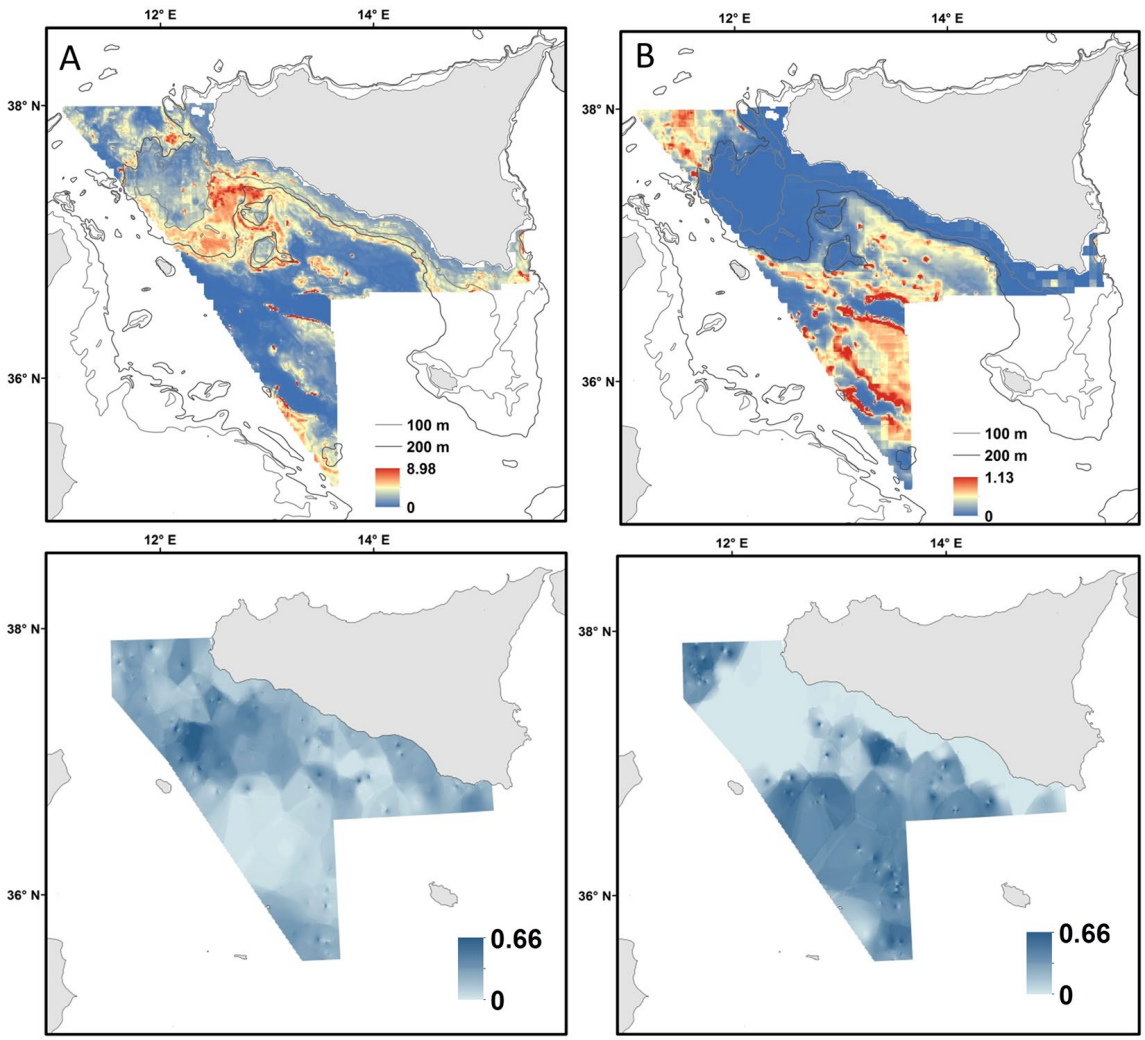
(**A**) *Funiculina quadrangularis* and (**B**) *Isidella elongata* in the Strait of Sicily. Predicted population densities from delta model (Nkm^−2^; top figures) representing preferential habitat, and associated prediction error (down Figures; 0 and 1 correspond to the minimum and maximum possible errors, respectively). These maps were created with ArcGIS version 10.3 http://www.esriitalia.it by Valentina Lauria.

The maps of model predictions of *F. quadrangularis* and *I. elongata* showed species-specific distribution patterns in response to diverse habitat requirements and are presented in Fig. 5. These revealed that *F. quadrangularis* prefers the shallow waters of the continental shelf and coastal areas (Fig. 5A), mostly corresponding to the Adventure bank and south-eastern coast of Sicily (Fig. 1). In contrast *I. elongata* seems to prefer deeper waters (Fig. 5B). The distribution patterns of these two octocorals species is rather different (Fig. 5) probably because their sensitivity to impact of fishing activities is diverse (see Fig. 2J and Figs 3 and 4). Model errors were calculated as the absolute difference between observed and predicted species abundance and mapped for the study area. In general, higher model uncertainty corresponded to areas of higher predictions (zones where species were caught regularly; Fig. 5).

## Discussion

This study enhances our understanding of the habitat requirements of and fishing impacts on two critically endangered deep-sea octocorals and provides the basis for the conservation of VMEs in the central Mediterranean Sea. Depth was an important factor influencing the habitat preferences of both octocoral species in the Strait of Sicily (Tables 3 and 5). The probability of presence of *F. quadrangularis* was higher at depths between 200–400 m, despite this species having a wider distribution range (20–2000 m; Table 1; Fig. 3). For *I. elongata* the probability of abundance was higher above 400 m depth (although this species favors depths between 200–800 m; Fig. 4). Our results are probably a regional characteristic of the Strait of Sicily, as generally both species can be found in deeper waters (Table 1). However, these results are partially limited by the fact that the MEDITS survey only reaches a maximum depth of 800 m, so it is difficult to know whether these species could be found at greater depths in this area. Our findings confirm other studies that have shown different depth preferences at a regional scale, for example in some parts of the Mediterranean Sea (Gulf of Lion) *F. quadrangularis* has been observed at an average depth of 239 m^22^, while in the North Atlantic it can be found at a depth range of 100–1200 m^20, 45^. Similarly *I. elongata* has been observed at different depths ranging from 680–750 m in the central and western Mediterranean Sea^71, 72^.

In the Strait of Sicily *F. quadrangularis* prefers areas identified by flat terrain that usually are characterised by soft bottoms (probability of abundance associated with low values of slope and rugosity; Tables 3 and 5; Fig. 3B), while the effect of these terrain variables was less marked on the habitat selection of *I. elongata* (Tables 3 and 5; Fig. 4B). This suggests that flat terrain soft-bottoms are not a limiting factor for *I. elongata* habitat selection. These results confirm the strong affinity previously observed between the distribution of pennatulaceans and fine sediments such as silt and clay^20, 73^. *Isidella elongata* appears to be mainly associated with compact slope muds^74^ even though it is also found on the sloping seafloor of marine canyons; probably because these areas cannot be reached by bottom-trawling and represent a refuge area for this vulnerable species^22^.

In our study the spatial distribution of *F. quadrangularis* seems to be negatively correlated to the seabed orientation (North-South direction) and currents while these relationships were positive for *I. elongata* (Figs 3 and 4; Table 5). Other studies on the spatial distribution of deep-sea corals have suggested the importance of seabed orientation and its exposure to currents^50, 53, 55^. In particular, deep-sea corals mounds were found to be more abundant in the central Mediterranean Sea associated with higher currents and consequently an increased nutrient supply^53^. In the Strait of Sicily *I. elongata* prefers substrata characterised by stronger currents (North-South direction; Fig. 4) probably due to local enrichment of food availability and low sediment rates^72^. In contrast, the spatial distribution of *F. quadrangularis* is associated with less exposed areas, as this species seems to prefer more stable environmental conditions^73, 74^. The possibility that the effect of currents on these species distribution patterns might vary locally cannot be ruled out; however, because of the broader resolution of the data used in this study (6.5 km) this relationship might not be fully explained in our model results.

The relationship with sea bottom temperature identified a species-specific optimum temperature range for the two octocorals in the Strait of Sicily. The probability of increased abundance of *F. quadrangularis* is higher at temperatures between 14–16 °C, while *I. elongata* seems to prefer temperatures between 13–14 °C (Figs 3 and 4; Table 5). In contrast, bottom salinity in the Strait of Sicily did not seem to constrain the spatial distribution of both species, despite an optimum range (38–38.5 PSU) being identified (Figs 3 and 4; Table 5). This specific preference for certain temperature and salinity ranges has been observed for other deep-sea coral species (such as *L. pertusa* and other sea pens in the North Atlantic^33, 46, 75^), which may be interpreted as a regional characteristic of the central Mediterranean Sea. Nonetheless, inference on the occurrence of local-scale effects in the study area is not allowed by current data limitation (resolution of 6.5 km), and further investigation at finer spatial resolution will be required.

Despite both *F. quadrangularis* and *I. elongata* are associated to fishing grounds of commercially valuable crustaceans (i.e. *P. longirostris*, *N. norvegicus*, *A. antennatus* and *A. foliacea* ^20–22^) the effect of fisheries on their probability of presence/abundance is quite different (Figs 3 and 4). In particular our results and predictive maps (Figs 2J and 5) suggest that while *F. quadrangularis* can be found in areas that are exploited by fisheries (positive curvilinear relationship between the probability of presence of *F. quadrangularis* and fishing effort; Fig. 3A), *I. elongata* is only present in areas where the fishing effort is low or absent (negative relationship between the probability of abundance and fishing effort). These species-specific responses to fishing impacts could be explained by their different body shapes (*F. quadrangularis* is pen-shaped while *I. elongata* is fan-shaped) and their structures for anchoring to the substrata. *Funiculina quadrangularis* attaches itself to the seafloor by using a bulb or peduncle that penetrates the sediment down to about 50 cm providing flexibility to the colony that can temporarily lie flat under the passage of fishing gear. Conversely *I. elongata* has a rooted holdfast embedded in the surface sediment and, due to its branched fan shape, a lower flexibility and a higher risk of being snagged by fishing gears (Table 1). Our results are supported by other findings where, contrary to expectations, sea pens were able to re-establish themselves in the sediments after fishing activities^76–78^, while the spatial distribution of *I. elongata* was strongly negatively correlated with the presence of bottom trawling suggesting that the optimal habitat of this species in the Mediterranean Sea has been significantly reduced^72, 79^.

## Conclusions

This study has important implications for the conservation of soft- and compact-mud VMEs in the central Mediterranean Sea as it represents the first attempt to identify key areas (Fig. 5). This enclosed basin has a unique marine fauna that includes several endemic species of deep-sea corals^80^. While some form of fisheries regulation has been proposed by the European Commission^81^ for these important deep-water ecosystems, its application is still ineffective in the Mediterranean, leaving the majority of its VMEs unprotected.

Deep-sea ecosystems are under-represented in the Marine Protected Areas of the Mediterranean Sea as the majority of these areas are coastal^82^. Following the recommendations of the GFCM (2006) for the establishment of fisheries-restricted areas and the protection of VMEs, some guidelines have been applied in the Mediterranean Sea^26^. However, they are only effective for certain species and regions (i.e. *Lophelia* Reefs of Santa Maria di Leuca; the area of cold hydrocarbon seeps off the Nile Delta; and the Eratosthenes Seamount). Another issue for the conservation of VMEs in the Mediterranean Sea is that the ban on trawling is only effective for species that are present below depths of 1000 m, leaving ecologically important deep-sea VMEs occurring at shallower depths unprotected. This includes the coral gardens formed by *I. elongata*, *F. quadrangularis* and other habitat-forming organisms such as crinoids and brachiopods^83^. The majority of the trawl fisheries in the Mediterranean deep waters targets highly prized crustaceans, such as rose and red shrimps and Norway lobsters, with significant impacts on non-target species, such as deep-sea corals. So far, in the Mediterranean Sea no specific measures have been taken to detect or map VMEs or to monitor the impacts caused by bottom fisheries^83^. Our results could be useful in implementing spatial conservation plans, informing the GFCM’s deep-sea fisheries, and protecting critically endangered deep-sea octocoral species, as well as their related VMEs, in the Mediterranean Sea. Further investigations into the habitat suitability for and spatial distribution of Mediterranean deep-sea corals will constitute a key requisite for informing policy makers and guaranteeing that fishing activities are compatible with conservation plans and objectives.

## Electronic supplementary material

Supplementary information
