# Supplementary material for: Species distribution models of two critically endangered deep-sea octocorals reveal fishing impacts on vulnerable marine ecosystems in central Mediterranean Sea
Source: Sci Rep. 2017 Aug 14;7:8049. doi: 10.1038/s41598-017-08386-z (PMC5556048; doi:10.1038/s41598-017-08386-z)
Supplement: Supplementary file 1 — Supplementary information [file 41598_2017_8386_MOESM1_ESM.pdf]

Supplementary Information for the research manuscript:

Species distribution models of two critically endangered deep-sea octocorals reveal fishing impacts on vulnerable marine ecosystems in central Mediterranean Sea

V. Lauria, G. Garofalo, F. Fiorentino, D. Massi, G. Milisenda, S. Piraino, T. Russo & M. Gristina

Table S1: Dates of MEDITS Survey in the Strait of Sicily from 2008 to 2013.

Year	Vessel	Start date	End date	Median day
2008	S. Anna	12-May-08	6-Jun-08	23/05
2009	S. Anna	21-May-09	16-Jun-09	03/06
2010	S. Anna	20-May-10	24-Jun-10	06/06
2011	S. Anna	24-Jun-11	02-Aug-11	05/07
2012	S. Anna	27-Jun-12	25-Jul-12	11/07
2013	S. Anna	08-Sep-13	09-Oct-13	23/09
